# Type II NKT Cells: An Elusive Population With Immunoregulatory Properties

**DOI:** 10.3389/fimmu.2018.01969

**Published:** 2018-08-28

**Authors:** Avadhesh Kumar Singh, Prabhanshu Tripathi, Susanna L. Cardell

**Affiliations:** Department of Microbiology and Immunology, Institute of Biomedicine, Sahlgrenska Academy, University of Gothenburg, Gothenburg, Sweden

**Keywords:** type II NKT cells, CD1d, sulfatide, T cell receptor, health and disease

## Abstract

Natural killer T (NKT) cells are unique unconventional T cells that are reactive to lipid antigens presented on the non-polymorphic major histocompatibility class (MHC) I-like molecule CD1d. They have characteristics of both innate and adaptive immune cells, and have potent immunoregulatory roles in tumor immunity, autoimmunity, and infectious diseases. Based on their T cell receptor (TCR) expression, NKT cells are divided into two subsets, type I NKT cells with an invariant TCRα-chain (Vα24 in humans, Vα14 in mice) and type II NKT cells with diverse TCRs. While type I NKT cells are well-studied, knowledge about type II NKT cells is still limited, and it is to date only possible to identify subsets of this population. However, recent advances have shown that both type I and type II NKT cells play important roles in many inflammatory situations, and can sometimes regulate the functions of each other. Type II NKT cells can be both protective and pathogenic. Here, we review current knowledge on type II NKT cells and their functions in different disease settings and how these cells can influence immunological outcomes.

## Introduction

Although conventional MHC-restricted T cells have been the main attraction for immunologists, unconventional T cells are continuously gaining increased attention. Despite of their low frequencies compared to conventional T cells, the subsets of unconventional T cells play an important role in various autoimmune diseases, cancers and infections, and are present both in human and mice. These cells include CD1d-restricted NKT cells, γδ TCR expressing T cells, and MR1-restricted mucosal associated invariant T cells (1). NKT cells are reactive to lipid antigens presented on the non-polymorphic MHC class I-like molecule CD1d (2). These cells are activated early in immune responses and can express immunoregulatory activities that determine different immune outcomes.

In contrast to conventional T cells that express highly diverse TCR, NKT cells appear comparatively limited in its TCR repertoire and ligand reactivities. Based on the nature of their TCR expression, NKT cells comprise two main categories, invariant or type I NKT cells, and diverse or type II NKT cells. Type I NKT cells, the most extensively studied subgroup, express a semi-invariant Vα14-Jα18 TCR in mice, and Vα24-Jα18 in humans paired with a limited repertoire of Vβ-chains (Vβ8.2, Vβ7, and Vβ2 in mice and Vβ11 in humans) (1, 3), while the less explored type II NKT cells utilize a more diverse TCR repertoire (4–7). In mice, type I NKT cells outnumber type II NKT cells,

while in humans the type II NKT cells are more frequent. Type I NKT cells are reactive to the marine sponge-derived glycolipid α-galactosylceramide (α-GalCer) (8), which forms a stable α-GalCer/CD1d tetramer reagent that can be used for detection of type I NKT cells (9, 10). Availability of α-GalCer/CD1d tetramers opened up new avenues of NKT cell research and laid the foundation for important findings in the field of immunoregulation. In contrast, type II NKT cells are not reactive to α-GalCer (11, 12), instead they are thought to make up an oligoclonal population that recognizes a diverse repertoire of lipid antigens (5, 7, 13). As ligand/CD1d tetramer reagents only recognize a fraction of type II NKT cells, and due to the lack of specific surface markers, it is currently not possible to identify the entire type II NKT cell population using single or combined reagents. Instead, type II NKT cells are often define by indirect approaches. One way of identifying this population is by their CD1d-reactivity and absence of the Vα14 (mouse) or Vα24 (human) TCR α-chains. In a few cases, ligands recognized by type II NKT cells can be loaded on CD1d-tetramers and used to identify subsets of the population (14). Type II NKT cells are abundant in liver of both mice (15) and human (16), although the frequency of the entire type II NKT cell population in different organs is still unknown. However, recent advances in the field have highlighted the importance of type II NKT cells in different diseases. In this review, we discuss recent progress in the study of type II NKT cells and their critical role in different disease settings.

## Antigens for type II NKT cells

MHC-II-deficient mice facilitated the discovery of type II NKT cells, when it was noted that while conventional CD4^+^ cells were absent, there was still a significant population of peripheral CD4^+^ cells (4). Among CD4^+^ T cell hybridomas developed from these mice, several were CD1d-autoreactive and they expressed different TCR. These and other type II NKT cell hybridomas were instrumental for the identification of different lipid antigens (5, 11, 15, 17–19). With the identification of more CD1d-restricted ligands for type II NKT cells, the ligands could be categorized into different subtypes - sphingolipids and glycerolipids or phospholipids (7, 20). As α-GalCer is not recognized by type II NKT cells, taken together, this suggests that type I NKT cells and type II NKT cells react to different sets of CD1d-presented antigens, consistent with their distinct TCR, although there are some ligands that are recognized by both NKT subsets.

Consistent with the CD1d-autoreactivity of many type II NKT cell hybridomas, several self lipids have been defined as ligands. Sulfatide, a well studied ligand for type II NKT cells, was first identified as a ligand for the type II NKT cell hybridoma XV19 expressing a Vα1/Vβ16 TCR (4, 15, 19). Subsequently, type II NKT cell reactivity to sulfatide has been shown by independently derived hybridomas (15, 19, 21). Structural analysis of the XV19 TCR in complex with CD1d and sulfatide revealed similarities to both TCR-MHC interactions and type I NKT TCR-CD1d (22, 23). A significant population of sulfatide/CD1d tetramer-reactive cells, around 0.2–4% in different organs, were identified in naive mice (15). Sulfatide reactive cells displayed diverse TCR with oligoclonal expansions, and preferentially used Vα3/Vα1-Jα7/Jα9 and Vβ8.1/Vβ3.1-Jβ2.7 TCR segments (24). While type I NKT cells possess germline-encoded CDR3 region of the TCRα chain, type II NKT cells demonstrated predominantly non-germline but also germline encoded CDR3 of TCRα and TCRβ chains (24). The substantial fraction of sulfatide/CD1d-reactive cells, and the different TCR used for CD1-restricted sulfatide reactivity emphasized the diverse but oligoclonal nature of the TCR of these cells. Using XV19 hybridoma reactivity, we demonstrated that lysosulfatide, which lacks the fatty acid chain, was the most stimulatory sulfatide isoform, followed by C24:1 and C24:0 (19). Surprisingly, mice genetically deficient in cerebroside sulfotransferase (CST^−/−^) or UDP-galactose ceramide galactosyltransferase (CGT^−/−^), key enzymes required in the synthesis of sulfatides, showed the presence of sulfatide reactive type II NKT cells (15, 24). These findings suggest that sulfatide is dispensable for the development of sulfatide-reactive type II NKT cells. Further, the sulfatide reactive XV19 hybridoma showed high autoreactivity to splenocytes from CST^−/−^ mice (19). These findings may be explained by reactivity to several ligands by one TCR, shown by the fact that the self-lipids β-glucosylceramide and β-galactosylceramide as well as self-phospholipids can activate the sulfatide reactive XV19 type II NKT cells (17, 25).

Using lipid loaded tetramers, significant populations of human and mouse type II NKT cells were identified reactive to β-glucosylceramide and glucosylsphingosine, lipids that accumulate in Gaucher's disease (26). Sequencing of TCRβ-chains of the human tetramer positive cells revealed diverse TCR. Lysophosphatidylcholine (LPC) is another ligand recognized by both murine and human type II NKT cells (25, 27, 28). LPC is also recognized by a few human type I NKT cells, but not by murine type I NKT cells (29–31).

Type II NKT cells have also been shown to react to ligands of microbial origin. Phosphatidylglycerol, diphosphatidylglycerol (or cardiolipin), and phosphatidylinositol from *Corynebacterium glutamicum* or *Mycobacterium tuberculosis* can activate various type II NKT, but not type I NKT, hybridomas, in a CD1d-dependent manner. The specificities of these hybridomas for different lipid antigens were distinct, albeit partially overlapping (18). In a subsequent study, a more potent phosphatidylglycerol antigen from *Listeria monocytogenes* was identified that had a structure distinct from previously identified mammalian or microbial variants, as it contains short, fully saturated anteiso fatty acid lipid tails (32). Type II NKT cells may not only recognize lipid antigens but a recent study suggests that they also can recognize hydrophobic peptides presented on CD1d (33, 34). A report by Nishioka et al. found that a rat type II NKT cell clone, reactive to vascular endothelial cells, could recognize a CD1d-presented hydrophobic peptide derived from sterol carrier protein 2, a protein implicated in intracellular lipid transfer.

Considering the relatively large populations of primary type II NKT cells that recognize identified lipid ligands, one may speculate that the number of antigens recognized by type II NKT cells is limited. On the other hand, a single type II NKT cell TCR can bind several different antigens. Further, the type II NKT cells that bind a given lipid/CD1d-tetramer have diverse but oligoclonal TCR. Therefore, type II NKT TCR show degeneracy for antigen recognition. Importantly, the TCR repertoire of type II NKT cells appears to largely overlap between mice and human.

## Modes of type II NKT cell activation

Having characteristics of both NK and T cells, NKT cells can respond to either innate (TCR-independent) or adaptive (TCR-dependent) stimulation. Type I NKT cells not only respond very rapidly to stimulation through TCR by secreting diverse cytokines but also to cytokines (IL-12, IL-18, and type I IFN) alone or produced by Toll-like receptors (TLR)-activated DCs, in the absence of TCR-engagement (35). However, information regarding the activation of type II NKT cells is still scarce. In contrast to type I NKT cells, lysophospholipid-reactive type II NKT cell activation was independent of IL-12 during hepatitis B virus (HBV) infection (28). A subset of type II NKT cells were shown to respond with partially CD1d-independent IFN-γ production when co-cultured with CpG stimulated DC (36). This implies TCR-independent activation of type II NKT cells on the one hand, but also suggests that TLR activation of DC may have upregulated type II NKT cell ligands on CD1d. It therefore seems feasible that like type I NKT cells, type II NKT cells are not limited to activation by TCR-engagement but can also be activated independently of TCR in a proinflammatory cytokine milieu. However, this needs to be directly addressed. Thus, type II NKT cells can be activated through the TCR by exogenous antigens, such as microbial lipids, or self-lipids that may be upregulated on CD1d in activated DC. Moreover, they can likely be activated indirectly by pathogen derived or endogenous TLR-ligands acting on DC, or by inflammatory cytokines independently of the TCR. It is likely that under most circumstances, both TCR-engagement and TCR-independent stimulatory signals contribute to type II NKT cell activation.

## Features of type II NKT cells

Type I and type II NKT cells share several features that makes them different from conventional T cells, but the two subsets often have distinct functions in specific immune reactions (5, 7, 13). Transcription factors play an important role in the development of MHC-restricted conventional T cells, and their combinations guide the functional profile of these cells. The transcription factor promyelocytic leukemia zinc finger (PLZF), induced by TCR signaling by agonist self-ligands after positive selection, is crucial for the development of type I NKT cells (37, 38). Type I NKT cells can be sub-grouped into distinct functional subsets based on the combinations of transcription factors such as PLZF, T-bet and RORγt: NKT1 (PLZF^low^T-bet^+^), NKT2 (PLZF^hi^T-bet^−^) and NKT17 (RORγt^+^) cells that secrete TH1-, TH2-, and TH17-like cytokine patterns, respectively, upon activation (39, 40). Whether type II NKT cells follow similar developmental pathways and can be sub-grouped in similar functional subsets is not yet clearly understood. Studies have found that at least a subset of type II NKT cells have a constitutive production of IL-4 like type I NKT cells, and these type II NKT cells could be identified as IL-4-reporter^+^ cells that did not bind the α-GalCer/CD1d-tetramer, or were present in mice lacking type I NKT cells (28, 36). These type II NKT cells exhibited similar developmental requirements as type I NKT cells - PLZF along with signaling lymphocyte activation molecule-associated protein (SAP) played a crucial role (36). The cells were PLZF^hi^ and had an activated phenotype (CD44^+^, CD62L^−^, CD69hi) comparable to type I NKT cells. In other studies, mouse and human type II NKT cells were described as PLZF^int^ and having a more resting phenotype (24, 26, 41, 42). In addition, several reports suggest that type I and type II NKT cells have different cytokine-producing capacities, and interestingly, that IL-13 may be more produced by type II NKT cells (15, 26, 27, 36, 41, 42). Taken together, studies using TCR transgenic mice, lipid/CD1d-tetramers, and IL-4-reporter mice show that type II NKT cells are diverse also in their phenotype, cytokine secretion and activation state. While they frequently express NKT cell characteristic markers such as PLZF, NK receptors and CD122, some type II NKT cells seem more similar to type I NKT cells while others have a resting phenotype or different functional characteristics. This is in line with the diverse activities that have been associated with type II NKT cells.

## Type II NKT cells in different diseases

### Multiple sclerosis (MS) and experimental autoimmune encephalomyelitis (EAE)

MS is a demyelinating autoimmune disease of the central nervous system (CNS) in which myelin-derived protein/lipid antigens are targets for autoreactive T cells. The myelin sheath is a rich source of sulfatide. Sulfatide-reactive T cells are more frequent in peripheral blood of MS patients than in healthy individuals (43), and CD1d-sulfatide reactive type II NKT cells, but not type I NKT cells, accumulate in the CNS in the experimental autoimmune encephalomyelitis (EAE) mouse model for MS (15). Thus, sulfatide-reactive type II NKT cells are thought to play a role in EAE, and *in vivo* administration of sulfatide at the time of EAE induction prevented the disease in a CD1d-dependent manner (15). Sulfatide-reactive type II NKT cells induced anergy of type I NKT cells, tolerized DCs and CNS microglia and inhibited the effector function of the pathogenic autoantigen-reactive CD4 T cells (15, 44).

### Type 1 diabetes (T1D)

T1D is an autoimmune disease in which autoreactive T cells target pancreatic β-cells of the Langerhans' islets. We have shown that type II NKT cells from 24αβ transgenic mice can protect NOD mice from disease, dependent on costimulators inducible costimulator (ICOS) and PD1 interactions (45, 46). Besides being a major lipid in the myelin sheath, different species of sulfatide are also present in pancreatic β-cells and could serve as ligands for type II NKT cells during T1D immunopathogenesis (47). Sulfatide/CD1d-tetramer positive cells were indeed enriched in pancreas-draining lymph nodes in non-obese diabetic (NOD) mice. Sulfatide administration to NOD mice reduced T1D incidence and islet-specific T cell responses by inducing secretion of the anti-inflammatory cytokine IL-10 from DCs (48, 49), however, protection from T1D in NOD mice by sulfatide was not found in a second study (50).

### Tumor immunity

A role for NKT cells in tumor immunity is well established; particularly for type I NKT cells (51). In many human cancers, low levels of circulating type I NKT cells correlate with a poor prognosis; therefore, these cells have been targeted in a series of clinical trials (52). In some cancer forms, type I and type II NKT cells play opposing roles; while type I NKT cells promote, type II NKT cells suppress tumor immunity. Immunosuppression by type II NKT cells was shown to be mediated by IL-13 production resulting in the activation of TGF-β-secreting Gr1^+^CD11b^+^ myeloid derived suppressor cells (MDSCs), which in turn suppressed tumor-specific CD8^+^ T cells or type I NKT cells (51). A similar scenario may be present in multiple myeloma patients, in which IL-13 secreting LPC/CD1d-tetramer positive type II NKT cells were several fold increased in peripheral blood (27). It may therefore be a way forward to target tumor immunosuppression by type II NKT cells in cancer treatment, as discussed in a recent review (53). However, the roles of NKT cells in tumor immunity are more complex, as both type I and II NKT cells can promote tumor immunity, and there are other cancers in which both type I or type II NKT cells can suppress tumor immunity, as recently discussed in more detail (53, 54). The factors that determine whether NKT cells promote or suppress tumor immunity are not well understood, but it is possible that NKT cell activation by CD1d-expressing tumor cells will favor immunosuppressive functions.

### Ulcerative colitis (UC)

UC is a one of two forms of inflammatory bowel disease (IBD) characterized by Th-2-driven mucosal inflammation and tissue destruction in the colon. In UC, sulfatide-reactive type II NKT cells producing IL-13 were increased and suggested to have a colitogenic role (55, 56). Interestingly, in a mouse model for UC, a similar function was attributed to IL-13 producing type I NKT cells that were required to induce the disease (57). However, in different mouse models for IBD, type I NKT cells were either pathogenic or protective (56). Microbial components are abundant in the intestine, which are likely to activate the NKT cells during IBD and compromised intestinal barrier function. 24αβ TCR transgenic mice with elevated CD1d expression spontaneously developed colitis but here IFN-γ and IL-17 were the main players, not IL-13 (58). Moreover, a recent study has suggested the involvement of type II NKT cells in dextran sulfate sodium-induced colitis in mice provided choline-deficient diet (59).

### Obesity

Obesity is a disease of low-grade adipose-tissue inflammation with a potential cancer risk (60). Both protective and pathogenic roles of type I NKT cells in obesity has been reported by different studies as these cells can either produce anti-inflammatory cytokines IL-4 and IL-10 or pro-inflammatory cytokines IFN-γ, in adipose tissues (61–65). However, type II NKT cells exacerbated diet-induced obesity, as deduced from mice that lack type I NKT cells compared with CD1d^−/−^ mice (lacking both cell types) (66). Similarly, another recent study indicated that type II NKT cells in ldlr^−/−^ mice promote spontaneous obesity, as ldlr^−/−^ or ldlr^−/−^CD1d^−/−^ mice are less obese and have less adipose tissue inflammation than ldlr^−/−^Jα18^−/−^ mice (67). By contrast, it was recently shown that sulfatide-induced type II NKT cells prevented high fat diet-induced obesity in mice by regulating adipose tissue inflammation, and their transfer into obese mice resulted in improved weight loss and glucose tolerance (68).

### Liver inflammation and hepatitis

Type I and type II NKT cells can play opposing roles in liver inflammation (69). Type II NKT cells are more frequent in human liver than type I NKT cells (16). In liver ischemic-reperfusion injury or a conanavalin A-induced hepatitis model, type I NKT cells were rapidly activated and elicited liver inflammation. This was inhibited by sulfatide-activated type II NKT cells through the activation of plasmacytoid DCs and the production of IL-12 and MIP-2 that induced anergy in type I NKT cells (70, 71). In another study, type II NKT cells inhibited alcohol-induced liver disease in a similar manner (72). Interestingly, sulfatide-reactive type II NKT cells that express IL-13Rα2 were detected in human liver and suggested to play a role in the protection from liver fibrosis (73). Thus, sulfatide activated type II NKT cells regulate pro-inflammatory type I NKT cells in liver inflammation.

### Infectious diseases

A role for NKT cells in infectious diseases is well established (74). In fact, as described above, bacteria and viruses can stimulate type I NKT cells without TCR engagement in an innate-like manner. Data suggest that also type II NKT cells can be activated by infections in an innate-like manner, without TCR stimulation. The two NKT subsets exert an opposing role in response to certain infectious agents. In *Trypanosoma cruzi* infected mice, contrary to type I NKT cells, type II NKT cells showed a proinflammatory effect by reducing the titers of pathogen-specific antibodies (75). By contrast, an opposing role was shown in *Schistosoma mansoni* infection where type II NKT cells skewed the profile to Th2 cytokine secretion with decreased IFN-γ, here type I NKT cells supported IFN-γ secretion (76). We have demonstrated a protective role of sulfatide-activated type II NKT cells in a murine model for *Staphylococcus aureus* sepsis accompanied with decreased TNF-α and IL-6 (77). Further, as mentioned earlier, type II NKT cells could be activated by the glycolipid components from *Corynebacterium glutamicum* or *Mycobacterium tuberculosis* (18) and phosphatidylglycerol from *Listeria monocytogenes* (32). Studies have also established a role for NKT cells in antiviral immune responses. Earlier reports have shown that CD1d- and NKG2D-mediated activation of type II NKT cells in HBV infection caused liver damage in mice (78, 79). However, it was more recently found that modified self-lipids such as phosphatidylethanolamine and lysophosphatidylethanolamine, produced during HBV infection, induced activation of liver type II NKT cells that enhanced anti-viral immune responses, demonstrating the dual role of type II NKT cells in this infection (28). Evidence for a protective role of type II NKT cells has also been shown in human immunodeficient virus-1 (HIV-1) infection, where sulfatide-induced type II NKT cells reduced viral replication in humanized severe combined immunodeficiency (SCID-Hu) mice (80), and induced type I NKT cell anergy (81).

### Graft vs. host disease (GVHD)

GVHD is a serious complication that can follow bone marrow transplantation as a treatment for hematological malignancies. Type II NKT cells have been implicated in the regulation of GVHD. Using a mouse GVHD model, it was shown that type II NKT cells in donor bone marrow protected recipient mice from GVHD by producing IFN-γ, which induced apoptosis of donor T cells, and IL-4, that deviated the immune response toward a protective Th2 type (82). In contrast, in this model, type I NKT cells did not have an effect. In this context it is interesting to note that type II NKT cells are more frequent than type I NKT cells in human bone marrow. Human bone marrow derived type II NKT cells displayed a TH2 cytokine profile and suppressed mixed lymphocyte reactions (83).

### Gaucher's disease (GD)

GD is an inherited metabolic disease caused by a deficiency of lysosomal glucocerebrosidase, characterized by progressive lysosomal storage of β-glucosylceramide and glucosylsphingosine (84) with an increased cancer risk (85). A functionally unique subset of type II NKT cells, reactive to β-glucosylceramide and glucosylsphingosine, was identified in wild type mice and healthy humans (26). These cells constitutively expressed a T-follicular helper phenotype and provided efficient B cell help. Strikingly, these cells were activated and expanded in human GD and its murine disease model, and it was speculated that they might contribute to the increased risk of B cell malignancy observed in GD (26).

## Conclusions and future perspectives

Studies so far have well documented the diverse roles of type II NKT cells in different immunological contexts. The picture that emerges is of a population with diverse functions and phenotypes, substantially more heterogeneous than type I NKT cells. Recent publications have further established that type II NKT cells are activated and potentially involved in several human diseases, while their targeting in mouse disease models have provided promising results. Type II NKT cells are activated by a range of lipid antigens, and although different type II NKT TCR recognize the same lipids, the TCR repertoire is oligoclonal in nature. One of the greatest challenges is to further define the entire population of type II NKT cells. The identification of additional ligands that can be used to identify type II NKT cells with CD1d-tetramers will be instrumental in this quest. Considering the recognition of several lipids by the same type II NKT cell TCR, it will be of importance to elucidate the role of ligand specificity vs. a more promiscuous CD1d-reactivity for type II NKT cell development and activation. The relative role of TCR mediated/adaptive activation and innate, TCR-independent/cytokine mediated stimulation will also be critical to determine. So far type I NKT cells have been targeted in several clinical trials. Considering that type II NKT cells are more abundant in humans, and have been shown to play an immunoregulatory role in several diseases, the type II NKT cells have a largely unexplored immunotherapeutic potential. We have great expectations that the coming years will see rapid progress in this field.

## Author contributions

AKS, PT, and SC made intellectual contribution to the work. AKS and SC wrote the text. PT critically read the manuscript.

### Conflict of interest statement

The authors declare that the research was conducted in the absence of any commercial or financial relationships that could be construed as a potential conflict of interest.

## References

[1] Godfrey DI Uldrich AP McCluskey J Rossjohn J Moody DB. The burgeoning family of unconventional T cells. Nat Immunol. (2015) 16:1114–23. 10.1038/ni.3298 26482978

[2] Godfrey DI MacDonald HR Kronenberg M Smyth MJ Van Kaer L. NKT cells: what's in a name? Nat Rev Immunol. (2004) 4:231–7. 10.1038/nri1309 15039760

[3] Kumar V Delovitch TL. Different subsets of natural killer T cells may vary in their roles in health and disease. Immunology (2014) 142:321–36. 10.1111/imm.12247 24428389 PMC4080948

[4] Cardell S Tangri S Chan S Kronenberg M Benoist C Mathis D. CD1-restricted CD4+ T cells in major histocompatibility complex class II-deficient mice. J Exp Med. (1995) 182:993–1004. 7561702 10.1084/jem.182.4.993 PMC2192275

[5] Rhost S Sedimbi S Kadri N Cardell SL. Immunomodulatory type II natural killer T lymphocytes in health and disease. Scand J Immunol. (2012) 76:246–55. 10.1111/j.1365-3083.2012.02750.x 22724893

[6] Marrero I Ware R Kumar V. Type II NKT Cells in inflammation, autoimmunity, microbial immunity, and cancer. Front Immunol. (2015) 6:316. 10.3389/fimmu.2015.00316 26136748 PMC4470258

[7] Macho-Fernandez E Brigl M. The extended family of CD1d-restricted NKT cells: sifting through a mixed bag of TCRs, antigens, and functions. Front Immunol. (2015) 6:362. 10.3389/fimmu.2015.00362 26284062 PMC4517383

[8] Kawano T Cui J Koezuka Y Toura I Kaneko Y Motoki K. CD1d-restricted and TCR-mediated activation of valpha14 NKT cells by glycosylceramides. Science (1997) 278:1626–9. 9374463 10.1126/science.278.5343.1626

[9] Benlagha K Weiss A Beavis A Teyton L Bendelac A. *In vivo* identification of glycolipid antigen-specific T cells using fluorescent CD1d tetramers. J Exp Med. (2000) 191:1895–903. 10.1084/jem.191.11.1895 10839805 PMC2213523

[10] Matsuda JL Naidenko OV Gapin L Nakayama T Taniguchi M Wang CR. Tracking the response of natural killer T cells to a glycolipid antigen using CD1d tetramers. J Exp Med. (2000) 192:741–54. 10.1084/jem.192.5.741 10974039 PMC2193268

[11] Gumperz JE Roy C Makowska A Lum D Sugita M Podrebarac T. Murine CD1d-restricted T cell recognition of cellular lipids. Immunity (2000) 12:211–21. 10.1016/S1074-7613(00)80174-0 10714687

[12] Makowska A Kawano T Taniguchi M Cardell S. Differences in the ligand specificity between CD1d-restricted T cells with limited and diverse T-cell receptor repertoire. Scand J Immunol. (2000) 52:71–9. 10.1046/j.1365-3083.2000.00754.x 10886786

[13] Rossjohn J Pellicci DG Patel O Gapin L Godfrey DI. Recognition of CD1d-restricted antigens by natural killer T cells. Nat Rev Immunol. (2012) 12:845–57. 10.1038/nri3328 23154222 PMC3740582

[14] Dhodapkar MV Kumar V. Type II NKT cells and their emerging role in health and disease. J Immunol. (2017) 198:1015–21. 10.4049/jimmunol.1601399 28115591 PMC5300729

[15] Jahng A Maricic I Aguilera C Cardell S Halder RC Kumar V. Prevention of autoimmunity by targeting a distinct, noninvariant CD1d-reactive T cell population reactive to sulfatide. J Exp Med. (2004) 199:947–57. 10.1084/jem.20031389 15051763 PMC2211873

[16] Exley MA He Q Cheng O Wang RJ Cheney CP Balk SP. Cutting edge: compartmentalization of Th1-like noninvariant CD1d-reactive T cells in hepatitis C virus-infected liver. J Immunol. (2002) 168:1519–23. 10.4049/jimmunol.168.4.1519 11823474

[17] Rhost S Lofbom L Rynmark BM Pei B Mansson JE Teneberg S. Identification of novel glycolipid ligands activating a sulfatide-reactive, CD1d-restricted, type II natural killer T lymphocyte. Eur J Immunol. (2012) 42:2851–60. 10.1002/eji.201142350 22777932

[18] Tatituri RV Watts GF Bhowruth V Barton N Rothchild A Hsu FF. Recognition of microbial and mammalian phospholipid antigens by NKT cells with diverse TCRs. Proc Natl Acad Sci USA. (2013) 110:1827–32. 10.1073/pnas.1220601110 23307809 PMC3562825

[19] Blomqvist M Rhost S Teneberg S Lofbom L Osterbye T Brigl M. Multiple tissue-specific isoforms of sulfatide activate CD1d-restricted type II NKT cells. Eur J Immunol. (2009) 39:1726–35. 10.1002/eji.200839001 19582739 PMC4212524

[20] Dasgupta S Kumar V. Type II NKT cells: a distinct CD1d-restricted immune regulatory NKT cell subset. Immunogenetics (2016) 68:665–76. 10.1007/s00251-016-0930-1 27405300 PMC6334657

[21] Jeon SB Yoon HJ Park SH Kim IH Park EJ. Sulfatide, a major lipid component of myelin sheath, activates inflammatory responses as an endogenous stimulator in brain-resident immune cells. J Immunol. (2008) 181:8077–87. 10.4049/jimmunol.181.11.8077 19018000

[22] Girardi E Maricic I Wang J Mac TT Iyer P Kumar V. Type II natural killer T cells use features of both innate-like and conventional T cells to recognize sulfatide self antigens. Nat Immunol. (2012) 13:851–6. 10.1038/ni.2371 22820602 PMC3442777

[23] Patel O Pellicci DG Gras S Sandoval-Romero ML Uldrich AP Mallevaey T. Recognition of CD1d-sulfatide mediated by a type II natural killer T cell antigen receptor. Nat Immunol. (2012) 13:857–63. 10.1038/ni.2372 22820603

[24] Arrenberg P Halder R Dai Y Maricic I Kumar V. Oligoclonality and innate-like features in the TCR repertoire of type II NKT cells reactive to a beta-linked self-glycolipid. Proc Natl Acad Sci USA. (2010) 107:10984–9. 10.1073/pnas.1000576107 20534460 PMC2890761

[25] Maricic I Girardi E Zajonc DM Kumar V. Recognition of lysophosphatidylcholine by type II NKT cells and protection from an inflammatory liver disease. J Immunol. (2014) 193:4580–9. 10.4049/jimmunol.1400699 25261475 PMC4201983

[26] Nair S Boddupalli CS Verma R Liu J Yang R Pastores GM. Type II NKT-TFH cells against Gaucher lipids regulate B-cell immunity and inflammation. Blood (2015) 125:1256–71. 10.1182/blood-2014-09-600270 25499455 PMC4335081

[27] Chang DH Deng H Matthews P Krasovsky J Ragupathi G Spisek R. Inflammation-associated lysophospholipids as ligands for CD1d-restricted T cells in human cancer. Blood (2008) 112:1308–16. 10.1182/blood-2008-04-149831 18535199 PMC2515141

[28] Zeissig S Murata K Sweet L Publicover J Hu Z Kaser A. Hepatitis B virus-induced lipid alterations contribute to natural killer T cell-dependent protective immunity. Nat Med. (2012) 18:1060–8. 10.1038/nm.2811 22706385 PMC3478098

[29] Fox LM Cox DG Lockridge JL Wang X Chen X Scharf L. Recognition of lyso-phospholipids by human natural killer T lymphocytes. PLoS Biol. (2009) 7:e1000228. 10.1371/journal.pbio.1000228 19859526 PMC2760207

[30] Gumperz JE Miyake S Yamamura T Brenner MB. Functionally distinct subsets of CD1d-restricted natural killer T cells revealed by CD1d tetramer staining. J Exp Med. (2002) 195:625–36. 10.1084/jem.20011786 11877485 PMC2193772

[31] Pei B Speak AO Shepherd D Butters T Cerundolo V Platt FM Diverse endogenous antigens for mouse NKT cells: self-antigens that are not glycosphingolipids. J Immunol. (2011) 186:1348–60. 10.4049/jimmunol.1001008 21191069 PMC3166644

[32] Wolf BJ Tatituri RV Almeida CF Le Nours J Bhowruth V Johnson D. Identification of a Potent Microbial Lipid Antigen for Diverse NKT Cells. J Immunol. (2015) 195:2540–51. 10.4049/jimmunol.1501019 26254340 PMC5030721

[33] Nishioka Y Yamaguchi M Kawakami A Munehiro M Masuda S Tomaru U. Type II Natural Killer T cells that recognize sterol carrier protein 2 are implicated in vascular inflammation in the rat model of systemic connective tissue diseases. Am J Pathol. (2017) 187:176–86. 10.1016/j.ajpath.2016.09.014 27863214

[34] Nishioka YM S Tomaru U Ishizu A. CD1d-Restricted Type II NKT Cells Reactive with endogenous hydrophobic peptides. Front Immunol. (2018) 9:548. 10.3389/fimmu.2018.00548 29599785 PMC5862807

[35] Kinjo Y Kitano N Kronenberg M. The role of invariant natural killer T cells in microbial immunity. J Infect Chemother. (2013) 19:560–70. 10.1007/s10156-013-0638-1 23846426 PMC3822041

[36] Zhao J Weng X Bagchi S Wang CR. Polyclonal type II natural killer T cells require PLZF and SAP for their development and contribute to CpG-mediated antitumor response. Proc Natl Acad Sci USA. (2014) 111:2674–9. 10.1073/pnas.1323845111 24550295 PMC3932886

[37] Kovalovsky D Uche OU Eladad S Hobbs RM Yi W Alonzo E. The BTB-zinc finger transcriptional regulator PLZF controls the development of invariant natural killer T cell effector functions. Nat Immunol. (2008) 9:1055–64. 10.1038/ni.1641 18660811 PMC2662733

[38] Savage AK Constantinides MG Han J Picard D Martin E Li B. The transcription factor PLZF directs the effector program of the NKT cell lineage. Immunity (2008) 29:391–403. 10.1016/j.immuni.2008.07.011 18703361 PMC2613001

[39] Lee YJ Holzapfel KL Zhu J Jameson SC Hogquist KA. Steady-state production of IL-4 modulates immunity in mouse strains and is determined by lineage diversity of iNKT cells. Nat Immunol. (2013) 14:1146–54. 10.1038/ni.2731 24097110 PMC3824254

[40] Wang Y Sedimbi S Lofbom L Singh AK Porcelli SA Cardell SL. Unique invariant natural killer T cells promote intestinal polyps by suppressing TH1 immunity and promoting regulatory T cells. Mucosal Immunol. (2018) 11:131–43. 10.1038/mi.2017.34 28401935 PMC5638666

[41] Stenstrom M Skold M Ericsson A Beaudoin L Sidobre S Kronenberg M. Surface receptors identify mouse NK1.1+ T cell subsets distinguished by function and T cell receptor type. Eur J Immunol. (2004) 34:56–65. 10.1002/eji.200323963 14971030

[42] Rolf J Berntman E Stenstrom M Smith EM Mansson R Stenstad H. Molecular profiling reveals distinct functional attributes of CD1d-restricted natural killer (NK) T cell subsets. Mol Immunol. (2008) 45:2607–20. 10.1016/j.molimm.2007.12.022 18304639

[43] Shamshiev A Donda A Carena I Mori L Kappos L De Libero G. Self glycolipids as T-cell autoantigens. Eur J Immunol. (1999) 29:1667–75. 10.1002/(SICI)1521-4141(199905)29:05<1667::AID-IMMU1667>3.0.CO;2-U 10359121

[44] Maricic I Halder R Bischof F Kumar V. Dendritic cells and anergic type I NKT cells play a crucial role in sulfatide-mediated immune regulation in experimental autoimmune encephalomyelitis. J Immunol. (2014) 193:1035–46. 10.4049/jimmunol.1302898 24973441 PMC4110642

[45] Duarte N Stenstrom M Campino S Bergman ML Lundholm M Holmberg D. Prevention of diabetes in nonobese diabetic mice mediated by CD1d-restricted nonclassical NKT cells. J Immunol. (2004) 173:3112–8. 10.4049/jimmunol.173.5.3112 15322171

[46] Kadri N Korpos E Gupta S Briet C Lofbom L Yagita H. CD4(+) type II NKT cells mediate ICOS and programmed death-1-dependent regulation of type 1 diabetes. J Immunol. (2012) 188:3138–49. 10.4049/jimmunol.1101390 22371394

[47] Buschard K Blomqvist M Osterbye T Fredman P. Involvement of sulfatide in beta cells and type 1 and type 2 diabetes. Diabetologia (2005) 48:1957–62. 10.1007/s00125-005-1926-9. 16143863

[48] Buschard K Hanspers K Fredman P Reich EP. Treatment with sulfatide or its precursor, galactosylceramide, prevents diabetes in NOD mice. Autoimmunity (2001) 34:9–17. 10.3109/08916930108994121 11681495

[49] Subramanian L Blumenfeld H Tohn R Ly D Aguilera C Maricic I. NKT cells stimulated by long fatty acyl chain sulfatides significantly reduce the incidence of type 1 diabetes in nonobese diabetic mice [corrected]. PLoS ONE (2012) 7:e37771. 10.1371/journal.pone.0037771 22649557 PMC3359325

[50] Rhost S Lofbom L Mansson J Lehuen A Blomqvist M Cardell SL. Administration of sulfatide to ameliorate type I diabetes in non-obese diabetic mice. Scand J Immunol. (2014) 79:260–6. 10.1111/sji.12157 24795987

[51] Robertson FC Berzofsky JA Terabe M. NKT cell networks in the regulation of tumor immunity. Front Immunol. (2014) 5:543. 10.3389/fimmu.2014.00543 25389427 PMC4211539

[52] Waldowska M Bojarska-Junak A Rolinski J. A brief review of clinical trials involving manipulation of invariant NKT cells as a promising approach in future cancer therapies. Cent Eur J Immunol. (2017) 42:181–95. 10.5114/ceji.2017.69361 28860937 PMC5573892

[53] Kato S Berzofsky JA Terabe M. Possible therapeutic application of targeting type II natural killer T cell-mediated suppression of tumor immunity. Front Immunol. (2018) 9:314. 10.3389/fimmu.2018.00314 29520281 PMC5827362

[54] Wang Y Cardell SL. The yin and yang of invariant natural killer t cells in tumor immunity-suppression of tumor immunity in the intestine. Front Immunol. (2017) 8:1945. 10.3389/fimmu.2017.01945 29375569 PMC5767593

[55] Fuss IJ Joshi B Yang Z Degheidy H Fichtner-Feigl S de Souza H. IL-13Ralpha2-bearing, type II NKT cells reactive to sulfatide self-antigen populate the mucosa of ulcerative colitis. Gut (2014) 63:1728–36. 10.1136/gutjnl-2013-305671 24515806 PMC4782805

[56] Liao CM Zimmer MI Wang CR. The functions of type I and type II natural killer T cells in inflammatory bowel diseases. Inflamm Bowel Dis. (2013) 19:1330–8. 10.1097/MIB.0b013e318280b1e3 23518808 PMC3694171

[57] Heller F Fuss IJ Nieuwenhuis EE Blumberg RS Strober W. Oxazolone colitis, a Th2 colitis model resembling ulcerative colitis, is mediated by IL-13-producing NK-T cells. Immunity (2002) 17:629–38. 10.1016/S1074-7613(02)00453-3 12433369

[58] Liao CM Zimmer MI Shanmuganad S Yu HT Cardell SL Wang CR. dysregulation of CD1d-restricted type ii natural killer T cells leads to spontaneous development of colitis in mice. Gastroenterology (2012) 142:326–34 e1–2. 10.1053/j.gastro.2011.10.030 22057113 PMC3267843

[59] Sagami S Ueno Y Tanaka S Fujita A Niitsu H Hayashi R. Choline deficiency causes colonic Type II natural killer T (NKT) cell loss and alleviates murine colitis under type I NKT cell deficiency. PLoS ONE (2017) 12:e0169681. 10.1371/journal.pone.0169681 28095507 PMC5241147

[60] Divella R De Luca R Abbate I Naglieri E Daniele A. Obesity and cancer: the role of adipose tissue and adipo-cytokines-induced chronic inflammation. J Cancer (2016) 7:2346–59. 10.7150/jca.16884 27994674 PMC5166547

[61] Lynch L Nowak M Varghese B Clark J Hogan AE Toxavidis V. Adipose tissue invariant NKT cells protect against diet-induced obesity and metabolic disorder through regulatory cytokine production. Immunity (2012) 37:574–87. 10.1016/j.immuni.2012.06.016 22981538 PMC4991771

[62] Ohmura K Ishimori N Ohmura Y Tokuhara S Nozawa A Horii S. Natural killer T cells are involved in adipose tissues inflammation and glucose intolerance in diet-induced obese mice. Arterioscler Thromb Vasc Biol. (2010) 30:193–9. 10.1161/ATVBAHA.109.198614 19910631

[63] Sag D Krause P Hedrick CC Kronenberg M Wingender G. IL-10-producing NKT10 cells are a distinct regulatory invariant NKT cell subset. J Clin Invest. (2014) 124:3725–40. 10.1172/JCI72308 25061873 PMC4151203

[64] Schipper HS Rakhshandehroo M van de Graaf SF Venken K Koppen A Stienstra R. Natural killer T cells in adipose tissue prevent insulin resistance. J Clin Invest. (2012) 122:3343–54. 10.1172/JCI62739 22863618 PMC3428087

[65] Wu L Parekh VV Gabriel CL Bracy DP Marks-Shulman PA Tamboli RA. Activation of invariant natural killer T cells by lipid excess promotes tissue inflammation, insulin resistance, and hepatic steatosis in obese mice. Proc Natl Acad Sci USA. (2012) 109:E1143–52. 10.1073/pnas.1200498109 22493234 PMC3358828

[66] Satoh M Andoh Y Clingan CS Ogura H Fujii S Eshima K. Type II NKT cells stimulate diet-induced obesity by mediating adipose tissue inflammation, steatohepatitis and insulin resistance. PLoS ONE (2012) 7:e30568. 10.1371/journal.pone.0030568 22383967 PMC3284453

[67] Subramanian S Goodspeed L Wang S Ding Y O'Brien KD Getz GS Deficiency of invariant natural killer T cells does not protect against obesity but exacerbates atherosclerosis in Ldlr(-/-) Mice. Int J Mol Sci. (2018) 19:510 10.3390/ijms19020510 PMC5855732 29419749

[68] Hams E Locksley RM McKenzie AN Fallon PG. Cutting edge: IL-25 elicits innate lymphoid type 2 and type II NKT cells that regulate obesity in mice. J Immunol. (2013) 191:5349–53. 10.4049/jimmunol.1301176 24166975 PMC3847854

[69] Kumar V. NKT-cell subsets: promoters and protectors in inflammatory liver disease. J Hepatol. (2013) 59:618–20. 10.1016/j.jhep.2013.02.032 23669283 PMC4086465

[70] Halder RC Aguilera C Maricic I Kumar V. Type II NKT cell-mediated anergy induction in type I NKT cells prevents inflammatory liver disease. J Clin Invest. (2007) 117:2302–12. 10.1172/JCI31602 17641782 PMC1913490

[71] Arrenberg P Maricic I Kumar V. Sulfatide-mediated activation of type II natural killer T cells prevents hepatic ischemic reperfusion injury in mice. Gastroenterology (2011) 140:646–55. 10.1053/j.gastro.2010.10.003 20950612 PMC3114423

[72] Maricic I Sheng H Marrero I Seki E Kisseleva T Chaturvedi S. Inhibition of type I natural killer T cells by retinoids or following sulfatide-mediated activation of type II natural killer T cells attenuates alcoholic liver disease in mice. Hepatology (2015) 61:1357–69. 10.1002/hep.27632 25477000 PMC4376634

[73] Paquin-Proulx D Greenspun BC Pasquet L Strunz B Aleman S Falconer K. IL13Rα2 expression identifies tissue-resident IL-22-producing PLZF(+) innate T cells in the human liver. Eur J Immunol. (2018) 48:1329–35. 10.1002/eji.201747334 29677387 PMC6733416

[74] Brigl M Brenner MB How invariant natural killer T cells respond to infection by recognizing microbial or endogenous lipid antigens. Semin Immunol. (2010) 22:79–86. 10.1016/j.smim.2009.10.006. 19948416

[75] Duthie MS Kahn M White M Kapur RP Kahn SJ. Critical proinflammatory and anti-inflammatory functions of different subsets of CD1d-restricted natural killer T cells during Trypanosoma cruzi infection. Infect Immun. (2005) 73:181–92. 10.1128/IAI.73.1.181-192.2005 15618153 PMC538963

[76] Mallevaey T Zanetta JP Faveeuw C Fontaine J Maes E Platt F. Activation of invariant NKT cells by the helminth parasite schistosoma mansoni. J Immunol. (2006) 176:2476–85. 10.4049/jimmunol.176.4.2476 16456008

[77] Kwiecinski J Rhost S Lofbom L Blomqvist M Mansson JE Cardell SL. Sulfatide attenuates experimental Staphylococcus aureus sepsis through a CD1d-dependent pathway. Infect Immun. (2013) 81:1114–20. 10.1128/IAI.01334-12 23340309 PMC3639588

[78] Baron JL Gardiner L Nishimura S Shinkai K Locksley R Ganem D. Activation of a nonclassical NKT cell subset in a transgenic mouse model of hepatitis B virus infection. Immunity (2002) 16:583–94. 10.1016/S1074-7613(02)00305-9 11970881

[79] Vilarinho S Ogasawara K Nishimura S Lanier LL Baron JL. Blockade of NKG2D on NKT cells prevents hepatitis and the acute immune response to hepatitis B virus. Proc Natl Acad Sci USA. (2007) 104:18187–92. 10.1073/pnas.0708968104 17991774 PMC2084318

[80] Sundell IB Halder R Zhang M Maricic I Koka PS Kumar V. Sulfatide administration leads to inhibition of HIV-1 replication and enhanced hematopoeisis. J Stem Cells (2010) 5:33–42. [jsc.2010.5.1.33]jsc.2010.5.1.33 20861926

[81] Fernandez CS Kelleher AD Finlayson R Godfrey DI Kent SJ. NKT cell depletion in humans during early HIV infection. Immunol Cell Biol. (2014) 92:578–90. 10.1038/icb.2014.25 24777308

[82] Kim JH Choi EY Chung DH. Donor bone marrow type II (non-Valpha14Jalpha18 CD1d-restricted) NKT cells suppress graft-versus-host disease by producing IFN-gamma and IL-4. J Immunol. (2007) 179:6579–87. 10.4049/jimmunol.179.10.6579 17982047

[83] Exley MA Tahir SM Cheng O Shaulov A Joyce R Avigan D. A major fraction of human bone marrow lymphocytes are Th2-like CD1d-reactive T cells that can suppress mixed lymphocyte responses. J Immunol. (2001) 167:5531–4. 10.4049/jimmunol.167.10.5531 11698421

[84] Liu J Halene S Yang M Iqbal J Yang R Mehal WZ. Gaucher disease gene GBA functions in immune regulation. Proc Natl Acad Sci USA. (2012) 109:10018–23. 10.1073/pnas.1200941109 22665763 PMC3382552

[85] Taddei TH Kacena KA Yang M Yang R Malhotra A Boxer M. The underrecognized progressive nature of N370S Gaucher disease and assessment of cancer risk in 403 patients. Am J Hematol. (2009) 84:208–14. 10.1002/ajh.21362 19260119 PMC3008404

